# Investigating the Effect of RACZ Epidural Neuroplasty With Ozone Injection in Patients With Chronic Radicular Lumbar Pain: A Randomized Controlled Clinical Trial

**DOI:** 10.1155/prm/7449536

**Published:** 2026-11-01

**Authors:** Alireza Khajehnasiri, Reza Atef Yekta, Nader Ali Nazemian Yazdi, Mahmoud Ganjifard

**Affiliations:** ^1^ Department of Anesthesiology Intensive Care and Pain Shariati Hospital Tehran University of Medical Sciences Tehran Iran tums.ac.ir; ^2^ Pain Research Center Neuroscience Institute Tehran University of Medical Sciences Tehran Iran tums.ac.ir; ^3^ Department of Anesthesiology and Critical Care Dr. Ali Shariati Hospital Tehran University of Medical Sciences Tehran Iran tums.ac.ir; ^4^ Department of Anesthesiology and Pain Medicine Amir A’lam Hospital Tehran University of Medical Sciences Tehran Iran tums.ac.ir; ^5^ Department of Anesthesiology and Critical Care Faculty of Medicine Birjand University of Medical Sciences Birjand Iran bums.ac.ir; ^6^ Pain Research Center Neuroscience Institute Imam Khomeini Hospital Complex Tehran University of Medical Sciences Tehran Iran tums.ac.ir

**Keywords:** chronic radicular lumbar pain, disability, numeric rating scale, ozone injection, RACZ epidural neuroplasty

## Abstract

**Background:**

Although the exact causes of chronic lumbar radicular pain are still unknown, potential causes include epidural fibrosis, arachnoiditis, mechanical failure, nerve root inflammation, and structural and degenerative changes to the spine.

**Objective:**

This study was conducted to assess the analgesic effect of using epidural ozone injection along with routine RACZ neuroplasty in patients with low back pain.

**Methods:**

This is a prospective randomized clinical trial conducted on 40 patients with chronic radicular lumbar pain allocated into two equal groups of RACZ caudal neuroplasty with and without ozone injection. Two groups of twenty people each were created from forty patients with chronic radicular lumbar pain who did not improve with conservative measures. In Group B, RACZ neuroplasty was performed, while in Group A, RACZ neuroplasty was administered along with epidural ozone injection. Before the intervention, baseline pain intensity and the degree of back pain‐related disability were determined using the Numeric Rating Scale (NRS) and Oswestry Disability Index (ODI), respectively. Twenty‐four hours, 1 month, and 3 months after the procedure, the NRS was recorded again. ODI was also assessed 1 month and 3 months after the procedure.

**Results:**

The mean NRS scores in the intervention group before, and 24 h, 1 month, and 3 months after the procedure were 8.80 ± 1.47, 4.45 ± 2.01, 5.25 ± 1.25, and 5.40 ± 2.16, respectively, and corresponding values in the control group were 8.70 ± 1.30, 4.60 ± 1.60, 5.70 ± 1.66, and 6.40 ± 1.64, respectively (Group effect: *F* = 0.784, *p* = 0.381 and Time effect: *F* = 81.910, *p* < 0.001). The mean scores of the ODI before, 1 month, and 3 months after the procedure were 48.60 ± 17.65, 40.75 ± 15.38, and 43.70 ± 18.50, respectively, in the intervention group, while in the control group, the respective values were 57.20 ± 15.67, 43.90 ± 19.92, and 48.75 ± 12.83. (Group effect: *F* = 1.246, *p* = 0.271 and Time effect: *F* = 24.496, *p* < 0.001).

**Conclusion:**

While the addition of ozone to RACZ neuroplasty showed no significant benefit in this study, these findings are limited by the modest sample size and short follow‐up. Further large‐scale, well‐powered trials are required to definitively establish the therapeutic role of ozone in this population.

**Trial Registration:** Iranian Registry of Clinical Trials (IRCT): IRCT20150724023315N3

## 1. Introduction

Nearly 85% of people experience either primary or secondary low back pain during their lifetime. In 85% of cases, no specific cause is discernible, a condition classified as nonspecific low back pain [1, 2]. A percentage of patients with low back pain face severe pain with moderate‐to‐severe disability, generally caused by dysfunctional intervertebral discs, facet joints arthropathy, nerve root irritation, dural adhesions around the nerve root, and injuries to ligaments, fasciae, and muscles. The mechanisms underlying pain include nerve root compression, vascular compromise, inflammation, and biochemical disturbance [3]^.^

Although the exact causes of chronic lumbar radicular pain are still unknown, potential causes include epidural fibrosis, arachnoiditis, mechanical failure, nerve root inflammation, and structural and degenerative changes to the spine [4].

Conservative modalities are usually unsuccessful for chronic pain after spinal surgery, and reoperation has a lower chance of being effective than the initial surgery. On the other hand, spinal cord stimulation (SCS) therapy is expensive and generally inaccessible.

Chronic lumbar radicular pain, persisting for more than 12 weeks and often accompanied by sensory or motor deficits, affects approximately 5.3% of men and 3.7% of women. Although 23%–48% of disc‐related cases improve spontaneously, about 30% continue to experience pain after 1 year, with 20% losing work capacity and 5%–15% eventually requiring surgery [5, 6].

Postoperative spine surgery pain may arise from unresolved preexisting pain, surgical complications, or later structural changes. The risk increases with each additional surgery, and prior surgery is the strongest predictor of recurrent pain. Epidural fibrosis occurs in about 44% of patients undergoing a second procedure and exceeds 60% with further operations. Chronic pain after spinal surgery is defined as persistent or recurrent axial or radicular pain despite anatomically successful surgery. Epidural adhesion is thought to be the cause of chronic pain following spine surgery in 20%–36% of instances. Lysis of adhesion (LOA) in the RACZ method involves the use of a flexible catheter, followed by the injection of steroids, hyaluronidase, and local anesthetics [7, 8].

Epidural adhesion is not exclusively seen in patients undergoing surgery, and the leakage of nucleus materials into the epidural space can also trigger an inflammatory and fibrotic response.

Reoperation for relieving fibrosis has resulted in discouraging outcomes, with a success rate of only 12%. One of the successful techniques to alleviate low back pain caused by epidural fibrosis is the percutaneous LOAs, bypassing the obstacles hindering drugs from reaching affected nerve roots. A study showed that this method was effective in ameliorating radicular back pain in 25% or even more of patients who were unresponsive to routine treatments. In another study, Manchikanti et al. performed a modified 1‐2‐day RACZ procedure instead of the 3‐day procedure, finding no significant difference between these procedures [3].

Epidural fibrosis does not occur exclusively after spinal surgery; it can happen following annular rupture, hematoma, infections, and intrathecal contrast material injection. Scars created following back surgeries predispose nerve roots to injury and interfere with the supply of nutrients to nerves through cerebrospinal fluid (CSF) and blood vessels, rendering them prone to compression and damage [3].

Medical ozone also has recognized anti‐inflammatory, analgesic, and immunomodulatory properties. These effects include reducing proinflammatory cytokines such as IL‐6 and TNF‐alpha, improving local microcirculation, and modulating the activity of nociceptive nerve endings. In cases where direct access to the disc is difficult because of degenerative changes or hyperosteogeny, peridiscal ozone administration may still produce therapeutic effects by targeting the surrounding neural and vascular structures. These findings provide a pathophysiological basis for the clinical benefits observed in patients treated with ozone [9].

Previous studies have examined RACZ epidural neuroplasty and ozone therapy separately with heterogeneous results, and there is insufficient evidence that adding ozone provides additional benefit. Therefore, this randomized clinical trial compared RACZ neuroplasty with and without adjunctive ozone injection in patients with lumbar radicular pain [10].

## 2. Methods

This study was a randomized, patient‐ and data analyst–blinded clinical trial. The Ethical Code (IR.TUMS.NI.REC.1401.085) and the (https://irct.behdasht.gov.ir/trial/68446) were prospectively registered before recruitment of the first participant. The research population included those admitted to two teaching hospitals (Shariati Hospital and Amir Alam Hospital, Tehran University of Medical Sciences, Tehran, Iran) due to low back pain and underwent caudal neuroplasty (RACZ procedure).

Inclusion criteria included people aged between 25 and 80 years, lumbar radicular pain (confirmed by EMG‐NCV) unresponsive to noninvasive treatments (including pharmaceutical and physical therapy) for at least three months, and pain score of 4 or higher in verbal Numeric Rating Scale (NRS) of 0–10 (0: *no pain at all*, 10: *the most severe pain imaginable*). Patients with acute trauma, spinal fractures, malignancies, rheumatologic diseases, psychological diseases, peripheral neuropathies, hyperthyroidism, G6PD deficiency, coagulopathies, systemic infections, urinary and fecal incontinence, and local low back and sacral infections, as well as pregnant women, were excluded from the study. Additionally, patients who declined to participate or who were unable to be contacted for any reason following the surgery were excluded. Patients were not restricted to a single structural etiology of radicular pain. MRI‐defined diagnoses, including disc herniation or spinal stenosis, as well as a history of previous lumbar spine surgery, were recorded when available but were not used as separate eligibility strata.

Patients who met the eligibility requirements were given a thorough description of the study’s goals and procedures, and if they agreed to participate, they were asked to sign an informed consent form. Patients were selected by convenience sampling and were divided into the intervention group and the control group using the block randomization method with Size 4 (using CREATE A RANDOMISATION LIST computer program; https://www.sealedenvelope.com/simple-randomiser/v1/lists). To ensure allocation concealment, sealed envelopes were used. A sequentially numbered envelope containing the group assignment was prepared for each participant. The envelope was opened only after the participant had been enrolled, and all baseline data had been collected. The allocation sequence was generated by an independent biostatistician using the Sealed Envelope randomization program. The biostatistician was not involved in patient enrollment, intervention delivery, or outcome assessment. Eligible participants were enrolled by a research assistant who was not involved in the randomization process or outcome assessment. After enrollment and collection of baseline data, group assignment was performed by an independent research staff member who opened the sequentially numbered sealed envelopes. This individual was not involved in patient care or outcome assessment. Patients and the data analyst were blinded to group allocation. Owing to the nature of the procedure, blinding of the physician performing RACZ was not feasible. Outcome measures were assessed by an independent assessor who was unaware of the assigned groups. As both groups underwent the same RACZ procedure and the only difference was the final injected agent administered without the patients’ knowledge, the participants were unable to distinguish ozone injection from the control treatment. In this study, the primary outcome was the change in pain intensity measured by the NRS. The secondary outcomes were disability related to low back pain assessed by the Oswestry Disability Index (ODI) and changes in analgesic consumption during follow‐up. The primary endpoint analysis was defined at 3 months after the intervention. Pain intensity was assessed at baseline, 24 h, 1 month, and 3 months after the procedure, while ODI was assessed at baseline, 1 month, and 3 months after the procedure. Analgesic consumption was also evaluated at 1 month and 3 months compared with the preoperative period.

At the beginning of the study, a demographic characteristics questionnaire along with a checklist of clinical parameters was completed by interviewing the patients or their companions or reviewing relevant medical records. Before starting the intervention, pain intensity (based on NRS) and low back pain–related degree of disability (based on ODI) were determined at the baseline. Following the procedure, the NRS pain score was recorded again at 24 h, 1 month, and 3 months after the procedure, and ODI was recorded 1 month and 3 months after the procedure as well. Also, following 1 month and 3 months of postoperation, any change in the consumption of analgesics was questioned compared to the preoperative period as “decrease or cessation,” “no change,” or “increase.”

Anticoagulant and antiplatelet medications were held (if medically allowable) before the procedure according to well‐distinguished guidelines. The coagulation status was evaluated based on clinical history and, if necessary, by relevant paraclinical tests. The neuroplasty procedure was performed in the operating room under aseptic conditions with the patient in the prone position, a pillow placed under her/his pelvis and a support beneath their ankles, and lower extremities internally rotated. American Society of Anesthesiologists (ASA) standard monitoring was applied for all patients. Venous access was established, followed by intravenous injection of midazolam and fentanyl for sedation. The initial doses of midazolam and fentanyl were determined as 1–2 mg (IV) and 50–100  μg (IV), respectively, based on the patient’s condition. These doses were readministered during the procedure, as needed, in order to ensure enough sedation as well as effective verbal communication with the patient.

After the administration of prophylactic antibiotic (1 g of intravenous cefazolin) and after standard preparation and draping of the operative site, the procedure was performed using the St.Cox caudal neuroplasty kit (Seawon Meditech Co., Ltd., South Korea). A 15‐G or 16‐G RACZ needle was inserted through the sacral hiatus under fluoroscopy guidance, and 1‐2 mL of contrast (Omnipaque 240 mg/mL) was then administered to ensure the correct positioning of the needle in the epidural space. The remaining contrast material was then injected up to 10 mL (in two aliquots) to view the adhesion site or sites, filling flaws, and the architecture of the epidural space. Then, the RACZ catheter (ST.Cox epidural catheter manufactured by Korea—over a spiral‐tipped guide‐wire) was introduced through the epidural needle and attempted to be directed through the ventrolateral epidural space toward the defect site or the area designated based on the patient’s symptoms, intraoperative epidurogram, and/or lumbosacral MRI (Figure 1). If the attempts to direct the catheter to the ventrolateral epidural space were unsuccessful, it was directed through the route with minimum resistance in order to avoid injury to vulnerable structures. Adhesions were recanalized (to the extent possible) using mechanical maneuvers, and then, the contrast material was reinjected to ensure successful mechanical adhesiolysis and to determine the correct catheter tip position within the epidural space.

**FIGURE 1:**
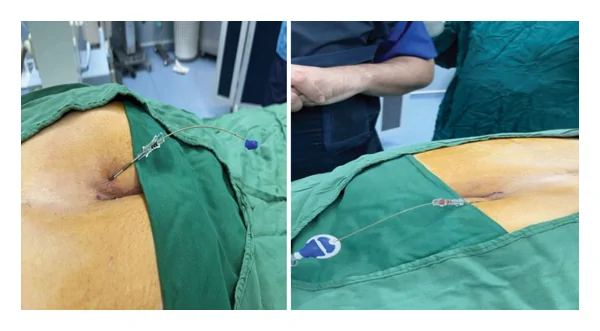
RACS procedure.

At each step of contrast material injection, in the case of vascular runoff, the catheter was pulled back, and the step was repeated, trying to redirect the catheter. A total of 1500 units of hyaluronidase dissolved in 10 mL of normal saline were slowly injected. After that, 3 mL of a 12‐mL mixture of 0.2% ropivacaine, and 40 mg methylprednisolone acetate was infused. In case of no evidence of intrathecal, subdural, or vascular distribution after 5 min (determined by sensory/motor block and fluoroscopic images), the rest of the admixture (up to 12 mL) was injected.

In Group A, 10 mL of ozone (25 μg/mL) was injected. Finally, after 15–20 min, 10 cc of 5% hypertonic saline solution was infused over 15 min. At the end, the catheter was removed (evaluating its intactness), and then, the needle was removed. The patient was monitored for vital signs and sensory/motor deficits for 2 hours and was finally discharged home after receiving the necessary education (Figure 2).

**FIGURE 2:**
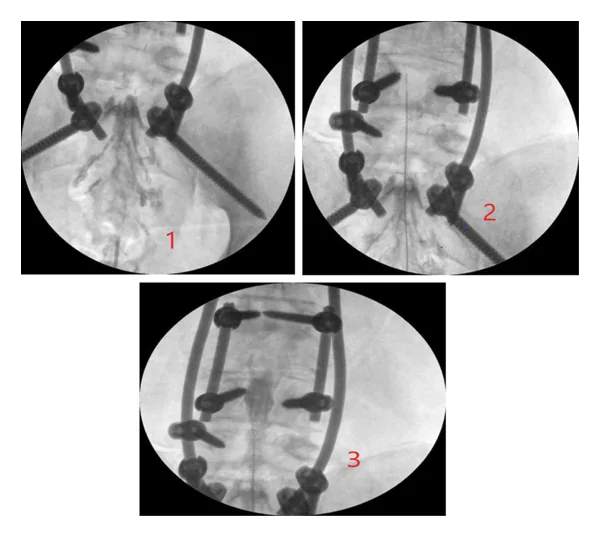
(1) Epidurography before catheter insertion; (2) catheter insertion and advancement after hyalase injection; (3) contrast injection after epidural adhesiolysis.

Demographic data of the patients along with duration of low back pain, onset, and location of back pain (back only, back and legs, and legs only), imaging findings (lumbosacral MRI), history of lumbar spine surgery, and epidurogram characteristics before and after neurolysis were recorded in previously prepared data sheets and were subjected to statistical analysis.

### 2.1. Data Collection Tools and Their Specifications

Data were collected through interviews, reviewing medical records, physical examination, and using specific questionnaires (appendix).

### 2.2. Sample Size

Based on a previous study entitled “Comparative effectiveness of paravertebral ozone injection and caudal epidural steroid hyaluronidase injection in lumbosacral spinal stenosis” and considering patients’ pain intensity in the epidural and ozone groups as 4.60 ± 1.12 and 5.70 ± 0.96, respectively, two weeks after the intervention, considering Type I error of 0.05, and study power of 90%, the sample size in each group was calculated as 19 patients. Taking into account a 20% attrition rate, 23 people were included in either group.

Following data collection, central tendency and dispersion statistics were used to characterize quantitative variables, while numbers and percentages were used to depict categorical variables. No prespecified subgroup analyses were performed according to MRI‐defined diagnosis, epidurogram findings, epidural fibrosis/adhesions, spinal stenosis, disc herniation, other degenerative changes, or history of previous lumbar spine surgery because the study was not powered for such comparisons.

### 2.3. Statistical Analysis

All analyses were performed according to the intention‐to‐treat (ITT) principle. Patients were analyzed in the groups to which they were originally randomized. As there were no protocol deviations or loss to follow‐up in either group (0% dropout rate), the ITT analysis was equivalent to the per‐protocol analysis. This approach preserves the benefits of randomization and avoids potential bias that could arise from excluding patients.

All analyses were based on per‐protocol design, including only completers. A between‐group comparison was assessed using the independent *t*‐test for quantitative variables and the chi‐square test for qualitative variables. Two‐way repeated measures analysis of variance (ANOVA) was performed to compare NRS and ODI across the two groups through the measurement times.

Normality assumption was assessed using the Shapiro–Wilk test for each group at each time point, and the assumption was met (all *p* > 0.05, ranged 0.121–0.789). Homogeneity of variances was confirmed using Levene’s test across all time points (all *p* > 0.05, ranged 0.230–0.653). Sphericity was evaluated using Mauchly’s test, which indicated that the sphericity assumption was met (Mauchly’s *W* = 0.753, *χ* ^2^ = 10.417, df = 5, *p* = 0.064 for NRS, and Mauchly’s *W* = 0.976, *χ* ^2^ = 0.886, df = 2, *p* = 0.642 for ODI). Therefore, no Greenhouse–Geisser correction was required. For all tests, the significance level was set at 0.05. (see Figure 3)

**FIGURE 3:**
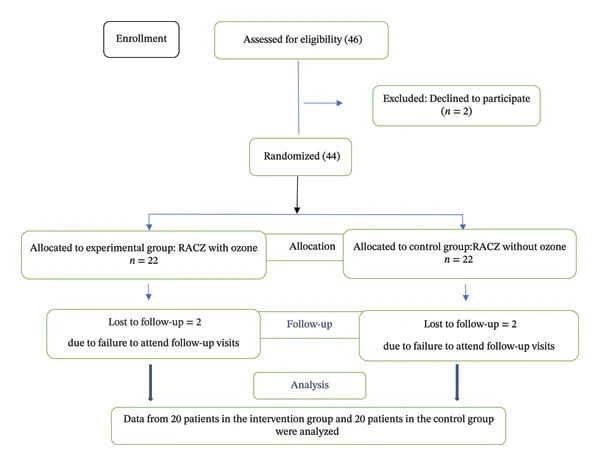
CONSORT flow diagram.

## 3. Results

This study was conducted to evaluate the effects of epidural neuroplasty with or without ozone infusion on chronic radicular lumbar pain. Two groups of patients (A: RACZ caudal neuroplasty with ozone injection, and B: RACZ caudal neuroplasty without ozone injection, *n* = 20 persons per group) participated in this trial. Most of the participants (80%) were women (75% in the intervention group and 85% in the control group); no significant difference was observed between the two groups in terms of gender (*p* = 0.695) (Table 1). The mean age of the participants in the intervention group was 55.80 ± 9.29 years compared to 58.85 ± 9.15 years in the control group, indicating no statistically significant difference between the two groups (*p* = 0.285).

**TABLE 1:** Demographic characteristics of patients according to the two studied groups.

Variable	Group			
	Intervention (*n* = 20)	Control (*n* = 20)	Total	*p* value
Age (yr)	55.80 ± 9.29	58.85 ± 9.15	57.33 ± 9.23	0.285
BMI (kg/m2)	26.82 ± 3.87	29.28 ± 3.92	28.05 ± 4.05	0.074
Gender				
Male	5 (25%)	3 (15%)	8 (20%)	0.695
Female	15 (75%)	17 (85%)	32 (80%)	
Changing the medication for 1 month				
Without change	16 (80%)	16 (80%)	32 (80%)	0.99
Decrease	4 (20%)	4 (20%)	8 (20%)	
Changing the medication for 3 months				
Without change	9 (45%)	13 (65%)	22 (55%)	0.543
Decrease	8 (40%)	5 (25%)	13 (32.5%)	
Additive	3 (15%)	2 (10%)	5 (12.5%)	

In the RACZ group, 11 patients (55%) had disc herniation at one or two levels, 4 patients (20%) had spinal stenosis, 2 patients (10%) had spinal canal narrowing due to other degenerative changes such as spondylosis or ligamentum flavum hypertrophy, and 3 patients (15%) had a history of previous lumbar spine surgery. In the RACZ plus ozone group, 12 patients (60%) had disc herniation at one or two levels, 3 patients (15%) had spinal stenosis, 2 patients (10%) had spinal canal narrowing due to other degenerative changes such as spondylosis or ligamentum flavum hypertrophy, and 3 patients (15%) had a history of previous lumbar spine surgery.

Table 2 indicates the results of two‐way repeated measures ANOVA to compare NRS pain score between the two groups during measurement times. As is shown in Table 2, group effect was not statistically significant (*p* = 0.381), meaning that there was no statistically significant difference in the mean NRS pain score between the intervention and control groups across measurement times as a whole (effect size = 0.020). Clearly, time effect was statistically significant (*p* < 0.001), meaning that there was a statistically significant change in the mean NRS pain score across measurement times in studied groups as a whole (effect size = 0.683). In addition, the group‐by‐time interaction effect was not statistically significant (*p* = 0.250), meaning that the slope of changes in mean NRS pain score across measurement times was not statistically differed between intervention and control groups (effect size = 0.035). In other words, the addition of epidural ozone injection did not make a statistically significant effect in reduction of NRS pain score compared to not addition of ozone injection. Figure 4 also depicts NRS pain score mean change during measurement times in intervention and control groups (Table 3).

**TABLE 2:** Comparison of NRS scores between the two groups at different times.

Time	Group			
	Intervention mean ± SD (95%CI) (*n* = 20)	Control mean ± SD (95%CI) (*n* = 20)	Mean difference (95% CI)	*p* value
Before	8.80 ± 1.47 (8.17, 9.43)	8.70 ± 1.3 (8.07, 9.33)	0.1 (−0.79, 0.99)	0.82
24 h after	4.45 ± 2.01 (3.63, 5.27)	4.60 ± 1.6 (3.78, 5.42)	−0.15 (−1.32, 1.02)	0.79
1 month after	5.25 ± 1.86 (4.45, 6.05)	5.70 ± 1.66 (4.9.6.49)	−0.45 (−1.58, 0.68)	0.42
3 months after	5.40 ± 2.16 (4.53, 6.27)	6.40 ± 1.64 (5.53, 7.27)	−1 (−2.23, 0.23)	0.11

**FIGURE 4:**
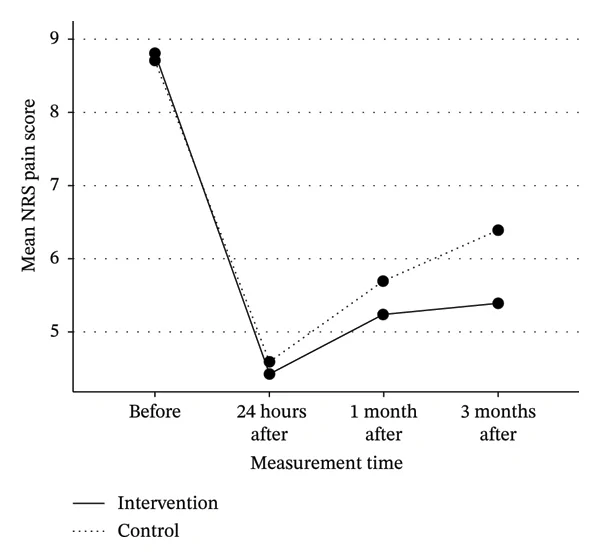
NRS pain score mean change through measurement times in intervention and control groups.

**TABLE 3:** Comparison of NRS pain score across the two groups and within‐subjects effects and between‐subjects effects.

Group effect	Time effect	Group ∗ time effect
*F* = 0.784	*F* = 81.910	*F* = 1.388
df = 1	df = 3	df = 3
*p* = 0.381	*p* < 0.001	*p* = 0.250
*η* ^2^ = 0.020	*η* ^2^ = 0.683	*η* ^2^ = 0.035

Results also demonstrated similar changes during the measurement times for NRS pain score and ODI means. Table 4 indicates the results of two‐way repeated measures ANOVA to compare ODI between the two groups during measurement times. Like NRS pain score, the group‐by‐time interaction effect was not statistically significant for ODI (*p* = 0.201, effect size = 0.041). In other words, the addition of epidural ozone injection did not make a statistically significant effect in improvement of ODI compared to not addition of ozone injection (Table 5). Figure 5 also depicts ODI mean change during measurement times in intervention and control groups.

**TABLE 4:** Comparison of Oswestry Disability Index (ODI) scores between the two groups at different times.

Time	Group			
	Intervention mean ± SD (95%CI) (*n* = 20)	Control mean ± SD (95%CI) (*n* = 20)	Mean difference (95% CI)	*p* value
Before	48.60 ± 17.65 (41.04, 56.16)	57.20 ± 15.67 (49.65, 64.76)	−8.6 (−19.29, 2.08)	0.11
1 month after	40.75 ± 15.38 (32.69, 48.81)	43.90 ± 19.92 (35.85, 51.96)	−3.15 (−14.54, 8.24)	0.58
3 months after	43.70 ± 18.50 (36.49, 50.91)	48.75 ± 12.83 (41.54, 55.96)	−5.05 (−15.24, 5.14)	0.32

**TABLE 5:** Comparison of Oswestry Disability Index (ODI) across the two groups and within‐subjects effects and between‐subjects effects.

Group effect	Time effect	Group ∗ time effect
*F* = 1.246	*F* = 24.493	*F* = 1.638
df = 1	df = 2	df = 2
*p* = 0.271	*p* < 0.001	*p* = 0.201
*η* ^2^ = 0.032	*η* ^2^ = 0.392	*η* ^2^ = 0.041

**FIGURE 5:**
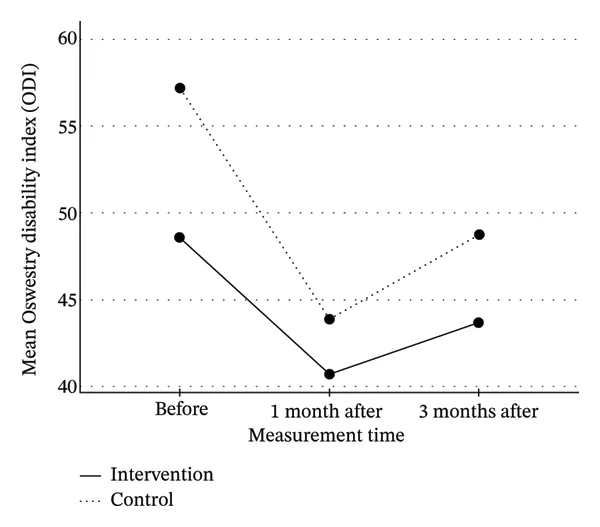
Oswestry disability index (ODI) mean change through measurement times in intervention and control groups.

## 4. Discussion

The present study showed that RACZ epidural neuroplasty significantly improved pain and disability in patients with chronic lumbar radicular pain; however, the addition of epidural ozone did not provide additional benefit. Rabinovitch et al. found a strong correlation between injection volume and improved results in a systematic review. Because of its high osmolality, which causes intracellular water to be expelled and reduces edema and inflammation, hypertonic saline has been shown to be more successful than normal saline in treating adhesive joint capsulitis. Usually, 6 mL of hypertonic saline is slowly injected over 30 min (in caudal neuroplasty). It is noticeable that if the dura is punctured (i.e., intrathecal injection), complications such as myelopathy, temporary or permanent paralysis, focal neurologic signs, or urinary incontinence may ensue [11]. Chronic low back and leg pain can result from conditions such as spinal stenosis, disc herniation, facet or sacroiliac joint disease, ligament injury, or postsurgical pain. When conservative treatments fail, percutaneous epidural neuroplasty is commonly used to manage chronic lumbar radicular pain [12, 13].

Ozone therapy has long been used for several decades since 1989 [14]. In that study, the patients had low back pain with various etiologies, whereas in the current study, the patients suffered from chronic lumbar radicular pain. The two studies also differ in terms of the injection site: In the present study, the injections were administered in the epidural space, while in the aforementioned study, they were performed paravertebrally and intradiscally. Therefore, given the differences in study design, patient population, route of administration, and the therapeutic role of ozone, the findings of the studies reviewed by Barbosa cannot be directly extrapolated to the clinical context of the present study.

Bonetti et al. compared intraforaminal ozone injection with periradicular steroid injection in 306 patients with acute or chronic back pain [15]. Therefore, differences in injection approach, type of therapeutic intervention, and study design may partly explain the discrepancy between the findings of Bonetti et al. and those of the present study and present randomized trial failed to demonstrate any additional therapeutic value of epidural ozone injection.

Manchikanti et al. evaluated epidural adhesiolysis in patients with chronic lumbar radicular pain who had poor response to epidural steroid injections. Patients receiving percutaneous epidural adhesiolysis with hypertonic saline showed substantial pain reduction (> 50%) in most cases at 3 and 6 months, with continued benefit in nearly half at 1 year, along with overall improvements in functional and health status compared with conservative treatment [3]. The evidence of this study suggests that the neuroplasty procedure itself can lead to significant and sustained pain improvement. This observation supports the interpretation that the clinical benefits seen in RACZ‐based interventions are likely primarily related to mechanical adhesiolysis and enhanced delivery of medications into the epidural space rather than the addition of ozone as a therapeutic agent. Accordingly, the absence of additional benefit in the ozone group in the present study is consistent with the hypothesis that epidural ozone does not provide a meaningful therapeutic advantage beyond the effect of the RACZ procedure itself.

Barbosa et al. assessed the impact of ozone therapy on pain and disability in patients with chronic pain after spinal surgery. They enrolled 19 patients who underwent epidural neuroplasty and ozone infusion and measured outcomes using several tools (i.e., Visual Analogue Scale (VAS), Brief Pain Inventory, and ODI). Patients reported significant improvement in pain as measured by VAS, but not in functional status measured by ODI, within 21 days after the procedure [1]. However, their study population consisted exclusively of postsurgical patients, whereas present trial included patients with chronic radicular pain regardless of prior surgery; therefore, the indication for injection differed between the two studies, which may partly explain the discrepancy in their results. Although the reviewed meta‐analysis reported a statistically significant benefit of ozone therapy for discogenic lumbosacral pain, the clinical interpretation of those findings requires caution. The pooled reduction in pain was notable; however, the included studies were highly heterogeneous regarding the route of ozone administration, dose and volume, concomitant treatments, follow‐up duration, and control interventions. Moreover, the meta‐analysis did not define or apply a prespecified minimal clinically important difference (MCID) for pain or disability outcomes. Therefore, while the overall findings support a potential analgesic effect of ozone in selected settings, they do not necessarily indicate that ozone provides additional clinical benefit when added to an already active interventional treatment. In contrast, the present study specifically evaluated the added value of epidural ozone injection in patients who all received caudal Racz neuroplasty. Both groups showed improvement in pain and disability over time, suggesting that the neuroplasty procedure itself was clinically effective. However, the addition of ozone did not produce statistically significant between‐group differences in NRS or ODI. From a clinical perspective, although both groups demonstrated clinically relevant within‐group pain reduction, the observed between‐group difference in NRS was small and did not appear to reach commonly used thresholds for a MCID. For ODI, improvement was not greater in the ozone group, indicating no clear functional advantage from adding ozone. Thus, the absence of statistical significance appears consistent with the lack of a clinically meaningful additional benefit. These findings do not contradict the meta‐analysis; rather, they suggest that the effectiveness of ozone may depend on the clinical context, comparator treatment, injection route, and whether ozone is used as a primary intervention or as an adjunct to a multicomponent procedure such as Racz neuroplast (1).

Considering the multimodal nature of the RACZ epidural neuroplasty procedure—including mechanical adhesiolysis, large‐volume epidural injection, and administration of local anesthetic, corticosteroid, hyaluronidase, and hypertonic saline—isolating the independent analgesic contribution of ozone is inherently challenging. The observed clinical improvements in both groups are, therefore, more likely attributable to the RACZ procedure itself rather than to the adjunctive use of ozone. The relatively small sample size of 40 participants (20 per group) represents an important limitation of this study. Although an a priori sample size calculation was performed based on a previous study, the obtained sample size may provide sufficient statistical power mainly for detecting large treatment effects. Therefore, the study may be underpowered to detect small‐to‐moderate differences between groups, increasing the possibility of a Type II error. Accordingly, the nonsignificant findings should be interpreted with caution.

### 4.1. Limitation

The present study has several limitations. First, due to the multimodal and extensive nature of the RACZ epidural neuroplasty procedure, including mechanical adhesiolysis, large‐volume epidural injection, and administration of local anesthetic, corticosteroid, hyaluronidase, and hypertonic saline, it was not possible to isolate the independent analgesic effect of ozone. These components are known to provide substantial pain relief on their own, which may have diluted any potential marginal effect of ozone. Second, adverse events were not systematically assessed using predefined criteria, and the follow‐up period was relatively short. The three‐month follow‐up period is a significant limitation; as chronic lumbar radicular pain requires longer‐term evaluation to assess the durability of treatment effects. Future studies should incorporate follow‐up periods of 6–12 months.

### 4.2. Suggestion

Although no major complications were observed during follow‐up, adverse events were not systematically assessed using predefined criteria. Given ozone’s oxidizing properties and ongoing concerns about its epidural safety profile, future studies should incorporate standardized adverse event monitoring and longer follow‐up.

## 5. Conclusion

In the present study, no statistically significant difference was observed between the two groups in terms of pain reduction or disability improvement. Although the addition of ozone did not demonstrate a significant advantage over RACZ epidural neuroplasty alone, these findings should be interpreted with caution given the modest sample size and limited follow‐up duration. Therefore, the results do not support a definitive conclusion regarding the ineffectiveness of ozone, and further studies with larger sample sizes and adequate statistical power are needed to better clarify its potential role.

## Author Contributions

Mahmoud Ganjifard: writing–original draft and methodology. Alireza Khajehnasiri: conceptualization, funding acquisition, review and editing, and methodology. Reza Atef Yekta: writing–original draft, review, and writing. Nader Ali Nazemian Yazdi: conceptualization, writing, and original draft.

## Funding

There is no funding for this study.

## Ethics Statement

The ethical code was obtained from Tehran University of Medical Sciences, IR.TUMS.NI.REC.1401.085. All methods were carried out in accordance with the Declaration of Helsinki, and informed consent was obtained from all the participants in the study.

## Consent

Informed consent was obtained from all eligible participants.

## Conflicts of Interest

The authors declare no conflicts of interest.

## Data Availability

The original contributions presented in the study are included in the article/supporting information; further inquiries can be directed to the corresponding author.
